# Supplementary material for: Glucose Regulates m6A Methylation of RNA in Pancreatic Islets
Source: Cells. 2022 Jan 15;11(2):291. doi: 10.3390/cells11020291 (PMC8773766; doi:10.3390/cells11020291)
Supplement: Supplementary file 1 [file cells-11-00291-s001.zip › cells-1407818-supplementary.pdf]

Article

Glucose Regulates m6A Methylation of RNA in Pancreatic Islets

Florine Bornaque [1], Clément Philippe Delannoy [1], Emilie Courty [1], Nabil Rabhi [1,†], Charlène Carney [1], Laure Rolland [1], Maeva Moreno [1], Xavier Gromada [1], Cyril Bourouh [1], Pauline Petit [2], Emmanuelle Durand [1], François Pattou [2], Julie Kerr-Conte [2], Philippe Froguel [1], Amélie Bonnefond [1], Frédérik Oger [1] and Jean-Sébastien Annicotte [1,*,‡]

[1] Institut Pasteur de Lille, University of Lille, Inserm, CHU Lille, CNRS, U1283-UMR 8199-EGID, F-59000 Lille, France; florine.bornaque@univ-lille.fr (F.B.); clement.delannoy@univ-lille.fr (C.P.D.); emilie.courty@univ-lille.fr (E.C.); nabil14006@gmail.com (N.R.); charlene.carney@cnrs.fr (C.C.); laure.rolland@univ-lille.fr (L.R.); maeva.moreno@inserm.fr (M.M.); gromada.xavier@gmail.com (X.G.); cyril.bourouh@univ-lille.fr (C.B.); emmanuelle.durand@cnrs.fr (E.D.); p.froguel@imperial.ac.uk (P.F.); amelie.bonnefond@cnrs.fr (A.B.); frederik.oger@univ-lille.fr (F.O.)

[2] University of Lille, Inserm, CHU Lille, U1190-EGID, F-59000 Lille, France; pauline.petit@univ-lille.fr (P.P.); francois.pattou@univ-lille.fr (F.P.); jkerr-conte@univ-lille.fr (J.K.-C.)

* Correspondence: jean-sebastien.annicotte@inserm.fr; Tel.: +33-(0)3-74-00-81-00

† Present address: Department of Biochemistry, Boston University School of Medicine, Boston, MA 02118, USA.

‡ Lead contact: Institut Pasteur de Lille, University of Lille, Inserm, CHU Lille, CNRS, U1283-UMR 8199-EGID, F-59000 Lille, France.

Citation: Bornaque, F.; Delannoy, C.P.; Courty, E.; Rabhi, N.; Carney, C.; Rolland, L.; Moreno, M.; Gromada, X.; Bourouh, C.; Petit, P.; et al. Glucose Regulates m6A Methylation of RNA in Pancreatic Islets. *Cells* **2022**, *11*, 291. https://doi.org/10.3390/cells11020291

Academic Editors: Gaetano Santulli

Received: 19 September 2021

Accepted: 12 January 2022

Published: 15 January 2022

Publisher's Note: MDPI stays neutral with regard to jurisdictional claims in published maps and institutional affiliations.

Copyright: © 2022 by the authors. Submitted for possible open access publication under the terms and conditions of the Creative Commons Attribution (CC BY) license (https://creativecommons.org/licenses/by/4.0/).

Running title: m^{6}A and glucose

Supplementary Data

Table S1. Islets Donor Information (Part. 1)

**Islet preparation**	**1**	**2**	**3**	**4**	**5**	**6**	**7**	**8**	**9**	**10**	**11**	**12**	**13**	**14**
**MANDATORY INFORMATION**														
Unique identifier	H596	H731	H785	H818	H834	H873	H879	H893	H900	H901	H1028	H1032	H1033	H1099
Donor age (years)	48	19	62	23	58	24	16	58	65	20	57	35	39	54
Donor sex (M/F)	H	F	H	H	F	F	F	H	F	H	F	M	M	F
Donor BMI (kg/m^2)	30,2	20,8	23,2	24,9	20,8	19,4	21,8	24,7	23,3	21,6	18.4	35.2	24.3	24
Donor HbA_{1c} or other measure of blood glucose control	5,6	5,6	5,4	5,4	5,6	5,9	5,1	5,2	5,6	5,6	6,1	5,4	5,6	5,8
Origin/source of islets[b]														
Islet isolation centre	LILLE	LILLE	LILLE	LILLE	LILLE	LILLE	LILLE	LILLE	LILLE	LILLE	LILLE	LILLE	LILLE	LILLE
Donor history of diabetes? Please select yes/no from drop down list														
**If Yes, complete the next two lines if this information is available**														
Diabetes duration (years)														
Glucose-lowering therapy at time of death[c]														
**RECOMMENDED INFORMATION**														
Donor cause of death	Traumatisme Non AVP	AVP	Traumatisme Non AVP	Traumatisme Non AVP	AVC	AVP	AVC	Anevrisme	AVC	AVP	AVC	AVC	Anoxia	Stroke

Warm ischaemia time (h)														
Cold ischaemia time (h)	5H35	3H24	5H59	9H51	6H55	3H58	6H31	3H05	10H33	4H00	4.22	4.57	3.07	7.03
Estimated purity (%)	70	80	60	80	90	80	80	55	90	50	90	80	80	90
Estimated viability (%)	95.8	96.3	90	95.2	97.6	98	93.4	98.7	93.5	87.5	86.9	95	96.2	96.2
Total culture time (h)[d]	12	19	43	20	18	18	21	20	21	20	16	18	18	60
Glucose-stimulated insulin secretion or other functional measurement[e]	Static : 1.82	Static : 1.37	Static : 0.78	Static : 0.96	Static : 1.06	Static : 1.69	Static : 0.93	Static : 1.14	Static : 1.17	Static : 1.51	Perifusion : 1.92	Perifusion : 7.19	Perifusion : 3.26	Perifusion : 4.74
Handpicked to purity? Please select yes/no from drop down list	No	No	No	No	No	No	No	No	No	No	No	No	No	No
Additional notes														

Table S1. Diabetic Islets Donor Information (Part. 2)

**Islet preparation**	**15**	**16**	**17**	**18**	**19**	**20**								
**MANDATORY INFORMATION**														
Unique identifier	H354	H504	H767	H774	H853	H894								
Donor age (years)	64	57	54	67	56	68								
Donor sex (M/F)	H	F	H	H	H	H								
Donor BMI (kg/m^2)	39,2	18,9	21,8	27,8	24,3	20,8								
Donor HbA_{1c} or other measure of blood glucose control	NA	NA	5,9	6,2	5,1	6,9								

Origin/source of islets[b]														
Islet isolation centre	LILLE	LILLE	LILLE	LILLE	LILLE	LILLE								
Donor history of diabetes? Please select yes/no from drop down list	NA	NA	NA	NA	NA	NA								
**If Yes, complete the next two lines if this information is available**														
Diabetes duration (years)														
Glucose-lowering therapy at time of death[c]														
**RECOMMENDED INFORMATION**														
Donor cause of death	Hemorragie Méningée	Anévrisme	AVC	Hematome Cérébral	AVC	Anoxie								
Warm ischaemia time (h)														
Cold ischaemia time (h)	4H00	15H12	5H58	6H56	3H35	5H20								
Estimated purity (%)	80	90	80	85	85	50								
Estimated viability (%)	75.8	96	90	97.4	88.5	NA								
Total culture time (h)[d]	18	23	20	18	18	22								
Glucose-stimulated insulin secretion or other functional measurement[e]	Static : 1.22	Static : 2.62	Static : 0.87	Static : 2.32	Static : 1.12	NA								
Handpicked to purity? Please select yes/no from drop down list	No	No	No	No	No	No								
Additional notes														

Supplementary Table S2. List of siRNA used for Min6 cells transfection

Target Gene	Species	Product name	Product Reference	Provider	Sequence
siRNA Control	Mouse	ON-TARGETplus Non-targeting Pool	D-001810-10-20	Dharmacon	UGGUUUACAUGUCGACUAA
					UGGUUUACAUGUUGUGUGA
					UGGUUUACAUGUUUUCUGA
					UGGUUUACAUGUUUUCCUA
siAlkbh5	Mouse	ON-TARGETplus SMARTpool siRNA Alkbh5	L-058321-01-0010, 268420, J-058321-09	Dharmacon	GAUUAGAUGCACCGCGAUU
			L-058321-01-0010, 268420, J-058321-10		GUUCAAGUUUGCUGCGUAU
			L-058321-01-0010, 268420, J-058321-11		GUUCAAGUUUGCUGCGUAU
			L-058321-01-0010, 268420, J-058321-12		GUUCAAGUUUGCUGCGUAU
siFto	Mouse	ON-TARGETplus SMARTpool siRNA Fto	L-062238-01-0010, 26383, J-062238-09	Dharmacon	CAACCUAAGUAACGGUAUA
			L-062238-01-0010, 26383, J-062238-10		CUGAAGUGUUGAAACGUUA
			L-062238-01-0010, 26383, J-062238-11		GUAGAAAGCAGUAGCGUUG
			L-062238-01-0010, 26383, J-062238-12		CGUCCGGACUUUACCAGUA
siMettl3	Mouse	ON-TARGETplus SMARTpool siRNA Mettl3	L-049446-01-0010, 56335, J-049446-09	Dharmacon	CAGCUAAGGAGCCGGCUAA
			L-049446-01-0010, 56335, J-049446-10		GAGGACUGUUUACGCAAUA
			L-049446-01-0010, 56335, J-049446-11		CUAAGAAUUUAUAGACGCA
			L-049446-01-0010, 56335, J-049446-12		AAGCUGCACUUCAGACGAA

Supplementary Table S3. List of oligonucleotides used in qPCR experiments

Official symbol	Species	Sequence	Forward / Reverse
CYPA (Cyclo)	Mouse	ATGGCACTGGCGGCAGGTCC	Forward
		TTGCCATTCCTGGACCCAAA	Reverse
	Human	ATGGCACTGGTGGCAAGTCC	Forward
		TTGCCATTCCTGGACCCAAA	Reverse
Alkbh5	Mouse	GCGCGGTCATCAACGACTA	Forward
		ATCAGCAGCATACCCACTGAG	Reverse
	Human	TGCAAGTTCCAGTTCAAGCC	Forward
		CTTGATGTCCTGAGGCCGTA	Reverse
METTL3	Mouse	CTGGGCACTTGGATTTAAGGAA	Forward
		TGAGAGGTGGTGTAGCAACTT	Reverse
	Human	ACAAGAAGCAGCTGGACTCT	Forward
		CAATGCTGCCTCTGGATTCC	Reverse
FTO	Mouse	TTCATGCTGGATGACCTCAATG	Forward
		GCCAACTGACAGCGTTCTAAG	Reverse
	Human	TTGGCTCCCTTATCTGACCC	Forward
		ACCAGGTCCCGAAATAAGCA	Reverse
Chop	Mouse	CTGCCTTTCACCTTGGAGAC	Forward
		CGTTTCCTGGGGATGAGATA	Reverse
	Human	GGAAACAGAGTGGTCATTCCC	Forward
		CTGCTTGAGCCGTTCATTCTC	Reverse
Xbp1s	Mouse	GAGTCCGCAGCAGGTG	Forward
		GTGTCAGAGTCCATGGGA	Reverse
	Human	CCGCAGCAGGTGCAGG	Forward
		GGGGCTTGGTATATATGTGG	Reverse
Atf4	Mouse	ATGGCCGGCTATGGATGAT	Forward
		CGAAGTCAAACTCTTTCAGATCCATT	Reverse
	Human	ATGACCGAAATGAGCTTCCTG	Forward
		GCTGGAGAACCCATGAGGT	Reverse
Atf6	Mouse	CATGTGGTGAATGTGCTGCC	Forward
		CACAGCGATATCCGAACCCA	Reverse
	Human	AGCAGCACCCAAGACTCAAAC	Forward
		GCATAAGCGTTGGTACTGTCTGA	Reverse
Pdx1	Mouse	ATTGTGCGGTGACCTCGGGC	Forward
		GATGCTGGAGGGCTGTGGCG	Reverse
Pax4	Mouse	GTGTACCCTCAGCTGCCTTG	Forward
		ATAGGCCTGGGATGAGGTGT	Reverse
MafA	Mouse	TCCGACTGAAACAGAAGCGG	Forward
		CTCTGGAGCTGGCACTTCTC	Reverse
Foxo1	Mouse	TGCCCAACCAAAGCTTCCCACA	Forward
		TGGACTGCTCCTCAGTTCCTGCT	Reverse
Gck	Mouse	GCTCAGTGAACCCCGGTCAGC	Forward
		TGTGCGCAGCTGCTCTGAGG	Reverse
Glut2	Mouse	AACCGGGATGATTGGCATGT	Forward
		GGCGAATTTATCCAGCAGCA	Reverse
Kir6.2	Mouse	CACAAGCTGGGTTGGGGGCTC	Forward
		TGCCCCTCAGCTGGGTTCTGC	Reverse
Ins1	Mouse	GCCAAACAGCAAAGTCCAGG	Forward
		GTTGAAACAATGACCTGCTTGC	Reverse
Ins2	Mouse	CAGCAAGCAGGAAGCCTATCT	Forward
		CAGGTGGGAACCACAAAGGT	Reverse

Supplementary Table S4. List of antibodies used for Dot Blot, Western Blot or Immunofluorescence experiments.

	Product Name	Species	Provider	Product Reference	Dilution
Primary Antibodies	Anti-Alkbh5 antibody	Rabbit	Abcam	ab195377	IF : 1/250<br>WB : 1/1000
	Anti-Mettl3 antibody	Rabbit	Abcam	ab195352	IF : 1/500<br>WB : 1/1000
	Anti-m6A antibody	Rabbit	Abcam	ab151230	Dot Blot : 1/500<br>IF : 1/100 (tissue)<br>IF : 1/250 (Min6)
	Anti-insulin antibody	Guinea-pig	Agilent technologies	A0564	IF : 1/500
	Anti-glucagon antibody	Mouse	Sigma	G2654	IF : 1/500
	Anti-α Tubulin antibody	Mouse	Abcam	ab7291	WB : 1/1000
	Anti-β Actin antibody	Mouse	Sigma	A5441	WB : 1/1000
	Anti-HDAC1 antibody	Rabbit	Abcam	Ab7028	WB : 1/1000
Secondary Antibody	Alexa Fluor 488 Anti-Rabbit antibody	Donkey	Invitrogen	A21206	1/200
	Alexa Fluor 564 Anti-Guinea Pig antibody	Goat	Invitrogen	A11075	1/200
	Alexa Fluor 647 Anti-Mouse antibody	Donkey	Invitrogen	A31571	1/200
	Anti-Mouse HRP antibody	Rabbit	Sigma	A9044	1/8000
	Anti-Rabbit HRP antibody	Goat	Sigma	A9169	1/8000

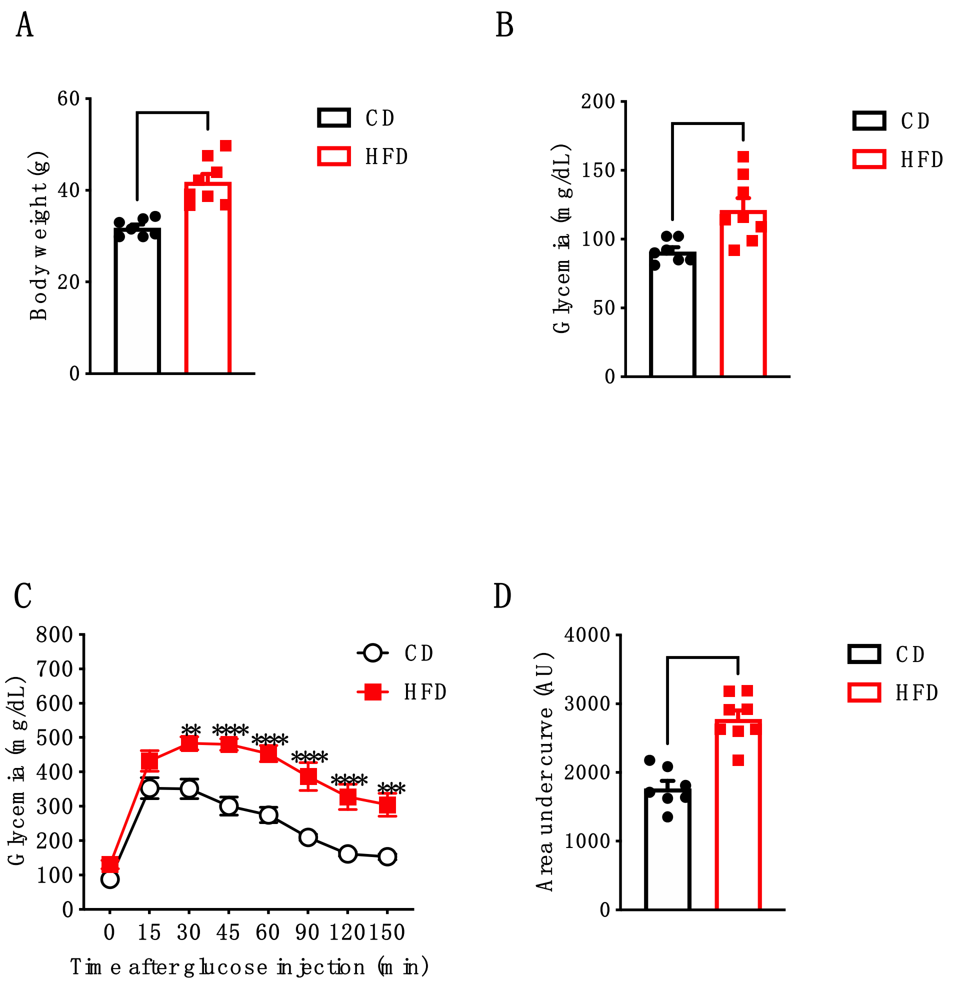

Supplementary Figure S1: Metabolic phenotyping of CD and HFD fed mice. (A) Body weight of C57Bl6J mice fed a regular chow (CD, n=7) or high fat diet (HFD, n=8). (B) Fasting blood glucose levels of C57Bl6J mice fed a regular chow (CD, n=7) or high fat diet (HFD, n=8). (C, D) Intraperitoneal glucose tolerance test (ipGTT, C) and the corresponding area under the curve of ipGTT (D) was performed after 16h fasting and glucose was administrated by intraperitoneal injection (2g/kg) in C57Bl6J mice fed a regular chow (CD, n=7) or high fat diet (HFD, n=8). Data were analyzed by Mann-Whitney tests (A, B, D) or 2way ANOVA with Tukey's correction for multiple comparisons (C) or. **p<0.01, ***p<0.001, ****p<0.0001.

Bornaque et al. supplementary Figure S2

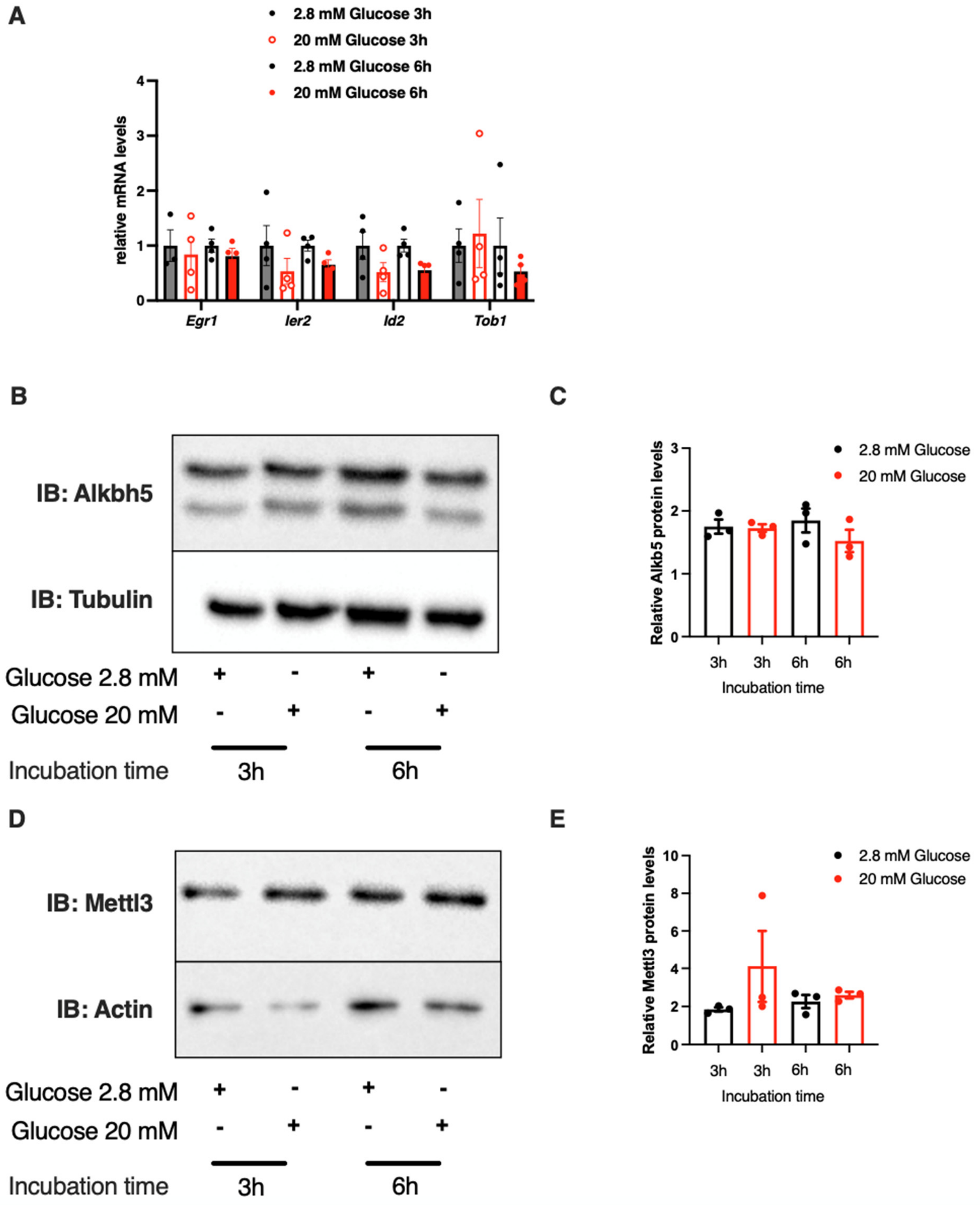

Supplementary Figure S2: mRNA levels of early glucose responder genes and protein levels of m6A reader and erasers after glucose treatment. (A) qPCR analysis of *Egr1*, *Ier2*, *Id2* and *Tob1* in Min6 cells treated for 3 and 6 hours with 2.8 mM and 20 mM of glucose. Individual values are presented (n=4). (B to E) Western blot assay (B, D) and its quantification (C, E) of ALKBH5 (B, C) and METTL3 (D, E) in Min6 cells exposed to 2.8 mM or 20 mM of glucose for 3 or 6 hours. Tubulin or actin were used as loading controls. Results in A, C and E are presented as mean+/- SEM.

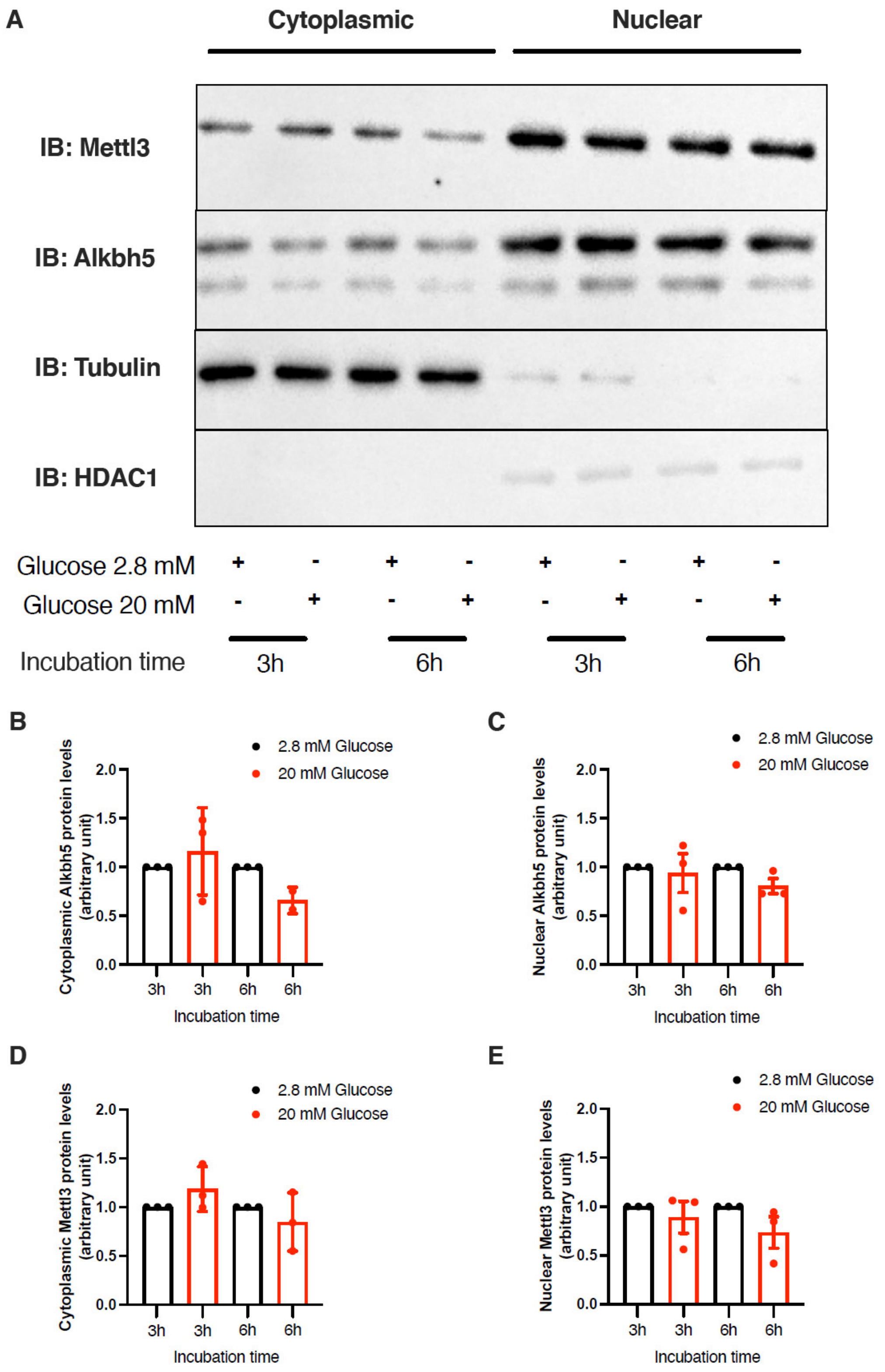

Supplementary Figure S3: protein localization of m6A reader and erasers after glucose treatment. Western blot assay (A) and its quantification (B to E) showing the nuclear and cytoplasmic localization of ALKBH5 and METTL3 in Min6 cells exposed to 2.8 mM or 20 mM of glucose for 3 or 6 hours. Tubulin or HDAC1 were used as loading controls for cytoplasmic and nuclear fractionation, respectively.
